# AI Chatbots in Chinese higher education: adoption, perception, and influence among graduate students—an integrated analysis utilizing UTAUT and ECM models

**DOI:** 10.3389/fpsyg.2024.1268549

**Published:** 2024-02-07

**Authors:** Weiqi Tian, Jingshen Ge, Yu Zhao, Xu Zheng

**Affiliations:** ^1^College of Foreign Languages, Xinjiang University, Urumqi, China; ^2^College of Liberal Arts, Journalism and Communication, Ocean University of China, Shandong, China

**Keywords:** AI Chatbot, Chinese graduate students, technology acceptance, UTAUT, ECM

## Abstract

This study is centered on investigating the acceptance and utilization of AI Chatbot technology among graduate students in China and its implications for higher education. Employing a fusion of the UTAUT (Unified Theory of Acceptance and Use of Technology) model and the ECM (Expectation-Confirmation Model), the research seeks to pinpoint the pivotal factors influencing students’ attitudes, satisfaction, and behavioral intentions regarding AI Chatbots. The study constructs a model comprising seven substantial predictors aimed at precisely foreseeing users’ intentions and behavior with AI Chatbots. Collected from 373 students enrolled in various universities across China, the self-reported data is subject to analysis using the partial-least squares method of structural equation modeling to confirm the model’s reliability and validity. The findings validate seven out of the eleven proposed hypotheses, underscoring the influential role of ECM constructs, particularly “Confirmation” and “Satisfaction,” outweighing the impact of UTAUT constructs on users’ behavior. Specifically, users’ perceived confirmation significantly influences their satisfaction and subsequent intention to continue using AI Chatbots. Additionally, “Personal innovativeness” emerges as a critical determinant shaping users’ behavioral intention. This research emphasizes the need for further exploration of AI tool adoption in educational settings and encourages continued investigation of their potential in teaching and learning environments.

## 1 Introduction

AI chatbots, such as OpenAI’s ChatGPT, utilize natural language processing and machine learning to simulate human-like conversations. ChatGPT, powered by a vast pre-trained dataset and reinforcement learning, demonstrates proficient language understanding, text generation, and high performance in diverse tasks like translation, composition, questioning, and summarization (Kasneci et al., 2023; Qadir, 2023). Since its launch in November 2022, ChatGPT has gained global attention for its human-like output quality, social capabilities, and extensive user base. Achieving 100 million active users within two months, it holds the record as the fastest-growing application in history (Harrison, 2023). The newest version, GPT-4, released in March 2023, extends its capabilities to image processing, significantly expanding its functionalities (Achiam, 2023).

The advent and ongoing progress of AI chatbots have catalyzed significant transformations across various fields, spanning education, finance, law, healthcare, media, and telecommunications (Aung et al., 2021). Particularly in education, the integration of large language models like ChatGPT has revolutionized the landscape, enabling personalized learning, cross-language learning methodologies, intelligent teaching strategies, efficient academic planning, and optimized management systems. They serve as essential aids for educators and virtual tutors for students, facilitating tasks like lesson planning, language translation, homework assignments, and essay composition (Qadir, 2023). Their versatility extends to addressing technical (e.g., engineering technology and computer programming) and non-technical issues (e.g., language and literature) across diverse disciplines. For example, in computer science, they provide explanations for various code, aiding students’ programming comprehension (MacNeil et al., 2022); in chemistry, they assist in evaluating answer quality (Moore et al., 2022); in language teaching, they offer personalized interaction, reducing learner anxiety (Bao, 2019); and in medical education, they aid in auto-grading, auxiliary teaching, information retrieval, and case-scenario generation (Thunström, 2022; Hamoudi, 2023).

Though AI Chatbots offer advantages in education, they have limitations such as potential inaccuracies, biases, lack of critical thinking, and ethical, privacy, and legal risks (Rahman and Watanobe, 2023; Zhu et al., 2023). Surprisingly, ChatGPT passed exams at the University of Minnesota Law School (Samantha, 2023). Concerns also exist about students misusing AI tools for plagiarism, exam cheating, and research data abuse (van Dis et al., 2023).

The emergence and ongoing evolution of AI Chatbots have catalyzed a global wave of AI innovation, with major industry players such as Google, Anthropic, and Meta launching their large language model products, such as Google’s Bard, Gemini, and Anthropic’s Claude. Simultaneously, in China, a multitude of generative AI tools have been introduced. Baidu unveiled “Wenxin Yiyan,” their large language model, in March 2023, while Alibaba initiated beta testing of “Tongyi Qianwen” in April, and Sensetime Technology released “Shangliang.” Additionally, various AI projects are in the pipeline, including JD Cloud’s “ChatJD,” Huawei’s “Pangu,” Tencent’s “Hunyuan,” and the Chinese Academy of Sciences Institute of Automation’s “Zidong Taichu.”

In China, highly educated young individuals form the primary cohort dedicated to the extensive use of AI chatbots, using new technologies to underscore their social status. Their adoption is fueled by this demographic emphasis. Furthermore, qualitative studies by Qiang and Hu (2023) have highlighted the allure of AI chatbots, identifying user attitudes as fervent admiration for innovation, objective acceptance of technological shocks, and understanding and tolerance for imperfections.

Chinese researchers acknowledge the dual nature of AI Chatbots, recognizing both their potential for human development and associated concerns (Hou and Li, 2023). These concerns include the potential for social biases, ethical issues, algorithmic enclosure, diminished user autonomy (Zhang and Jia, 2023), as well as privacy infringement, data security risks, and challenges related to intellectual property rights (Tan, 2023).

Prior research primarily focuses on AI Chatbot acceptance among undergraduate students, with limited attention to graduate students, particularly in China. Given that graduate students are essential to scientific research and innovation, and their research progress holds significant societal implications, this study aims to explore the acceptance and usage of AI Chatbots among Chinese graduate students, addressing this literature gap:

1. What is the factorial structure of the acceptance and utilization of AI Chatbot technology among graduate students in China, considering the combined framework of the UTAUT model and the ECM model?
2. What are the structural relationships among the predictors of graduate students’ attitudes, satisfaction, and behavioral intentions (BI) towards AI Chatbots in the context of higher education in China?

## 2 Literature review

### 2.1 The use of AI Chatbot in higher education

The emergence and progression of AI chatbots as technological constructs signify a substantial advancement in the realm of human-computer interaction. As postulated by Xu et al. (2020), AI chatbots embody sophisticated systems capable of assimilating real-time data from diverse sources to provide customized responses, suggestions, and resolutions to complex customer inquiries. This functional capacity is deeply rooted in the historical underpinnings of AI chatbots, dating back to the seminal inquiry posed by Alan Turing in the 1950s, “Can machines think?”—a foundational question within the field of AI, as extensively documented by Medina and Ferrer (2022).

The evolution of AI chatbots has significantly revolutionized customer service, offering real-time, personalized support by integrating data from various touchpoints, thus enhancing customer engagement and satisfaction. Through constant learning and adaptation, AI chatbots can better understand and respond to the nuanced needs and preferences of users, reshaping the landscape of human-computer interaction and challenging traditional models of customer engagement. This versatility and adaptability underscore the transformative power of AI chatbots, positioning them as pivotal assets in reshaping the dynamic between technology and human interaction.

The field has seen the emergence and persistent use of various chatbots, with contemporary models like ChatGPT and those developed by Chinese entities showcasing advanced features such as voice recognition and synthesis. These advancements significantly expand the practical scope of chatbots across a wide array of industries, including the crucial domain of education and language acquisition.

In education, AI chatbots are gaining prominence as dynamic instructional agents, proficient in delivering educational content, engaging learners in dialogue, and providing instant feedback, as observed by Mageira et al. (2022). Their role is notably complementary to that of human educators, offering continuous assistance and support to students—a function that has gained significant importance in the context of remote learning and language acquisition. This dualistic role has been explored by scholars such as Shawar (2017), who have identified the potential of chatbots in alleviating language-related anxiety, enhancing student engagement, and facilitating iterative learning opportunities enriched by multimodal interactions.

The research conducted by Wu and Yu (2023), employing meta-analytical methodologies to explore the influence of AI chatbots on educational outcomes, confirms the considerable efficacy of chatbots within higher education, in contrast to the more modest effects observed in primary and secondary education settings. However, the incorporation of chatbots in education is not without its challenges. Notably, the work of Wang et al. (2023) highlights prominent concerns related to privacy, cultural considerations, and language proficiency barriers, offering a critical examination of AI’s integration into educational administration and pedagogy.

Meanwhile, ChatGPT represents a novel chatbot deeply rooted in the Generative Pre-training Transformer architecture. Extensive analyses by Dwivedi et al. (2023), Farrokhnia et al. (2023), Su et al. (2023), and Tlili et al. (2023) confirm its superiority over early chatbots in the realms of understanding and generating human-like texts, and providing comprehensive feedback on lengthy textual inputs. These distinctive capabilities position ChatGPT as a formidable writing assistant and tool, as supported by research from Barrot (2023), Dergaa et al. (2023), and Imran and Almusharraf (2023). Furthermore, Taecharungroj’s (2023) analysis of early reactions on Twitter underscores ChatGPT’s predominant use in writing applications such as the composition of essays and articles. This widespread recognition and adoption of ChatGPT within writing tasks signal its instrumental role in facilitating and enhancing the writing process.

The collective evidence underscores ChatGPT’s transformative impact on writing and its substantial advancement over previous chatbot models. Its sophisticated text generation capabilities have positioned it as an invaluable resource in assisting writers across various domains, thereby underscoring the influential role it plays in shaping the landscape of writing and content generation. Assessing the scholarly discourse on ChatGPT, Dergaa et al. (2023) and Imran and Almusharraf (2023) underscore the necessity of harnessing ChatGPT as a valuable writing assistant tool in bolstering the writing process and enriching academic composition. Their contributions spotlight the pivotal role that ChatGPT assumes in elevating the quality of academic writing and streamlining the writing process within academic settings.

While the preceding user cases and academic discourse provide valuable insights into the potentials and challenges of integrating AI chatbots in education, it is critical to note that research in this area is still in its nascent phase (Barrot, 2023). Empirical research investigating the socio-technical dimensions of utilizing AI chatbots in higher education remains limited. There exists a need to delve into and assess the intention of postgraduate students regarding AI chatbots in learning and research, in addition to examining the influential factors at play. Such investigations hold the potential to elucidate postgraduate students’ acceptance of AI chatbots in educational and research contexts, offering valuable insights to leverage AI chatbots effectively within higher education.

Amid this landscape, it becomes evident that there is a pressing need to advance empirical research focusing on the integration of AI chatbots in higher education. Understanding postgraduate students’ perceptions and intentions towards AI chatbots can offer nuanced insights into the potential benefits and challenges associated with their utilization, thereby guiding the strategic integration of AI chatbots to augment the scholarly landscape within higher education. This underscores the necessity for further, in-depth investigations to not only understand postgraduates’ readiness to adopt AI chatbots but also to identify effective strategies to harness AI chatbots in advancing research and learning pursuits within higher education. Through such explorations, educators and researchers can gain critical insights essential for the effective leveraging of AI chatbots as pedagogical aids and in scholarly research within the higher education domain.

### 2.2 UTAUT

In 2003, Venkatesh and others constructed the Unified Theory of Acceptance and Use of Technology (UTAUT) model based on the integration and expansion of eight theories and models, including the Theory of Reasoned Action (TRA) and the Technology Acceptance Model (TAM). The UTAUT model introduces a more comprehensive theoretical framework to understand user technology acceptance and usage behavior by consolidating four core variables (Venkatesh et al., 2003, 447–453):

1. Performance Expectancy (PE): This refers to “the degree to which an individual believes that using the system will help him or her to attain gains in job performance”;
2. Effort Expectancy (EE): This refers to “the degree of ease associated with the use of the system”;
3. Social Influence (SI): This refers to “the degree to which an individual perceives that important others believe he or she should use the new system.”
4. Facilitating Conditions (FC): This refers to “the degree to which an individual believes that an organisational and technical infrastructure exists to support the use of the system.”

Moreover, four moderating variables were introduced: gender, age, experience, and voluntariness (Venkatesh et al., 2003).

While TAM models explain at most 40% of Behavioral Intentions (BI), UTAUT explains up to 70% of variability in user acceptance and usage intention (Holden and Karsh, 2009).

Multiple studies applied the UTAUT model to various digital landscapes.

1. In E-learning, PE, SI, and FC directly influenced students’ attitudes towards the usage of Moodle, with PE as the strongest determinant of students’ attitude (Šumak et al., 2010).
2. In E-Government, significant positive correlations were found between FC, PE, EE, SI, System Trust, and Net Ethics, influencing the Behavioral Intentions (BI) to use e-government services. Age, education level, and the BI to use e-government services have significant differences, excluding gender (Zeebaree et al., 2022).
3. In M-payment, the best predictor of the intention to use a mobile payment system was PE, followed by SI, EE, Perceived Trust, Perceived Cost, and Self-efficacy (Al-Saedi et al., 2020).
4. In AI, the correlation was found to be significantly positive between PE and BI to utilize AI in recruitment, with no significant impact discovered in terms of gender, age, experience, and education level (Horodyski, 2023).
5. In AI-assisted education, EE, PE, and SI were positively correlated with university students’ usage of AI-assisted learning, with Psychological Risk being a significant negative influence on the students’ BI (Wu et al., 2022).
6. In Web-based learning, PE, EE, Computer Self-efficacy, Achievement Value, Utility Value, and Intrinsic value were significant predictors of individuals’ intention to continue using web-based learning. Anxiety has a significant negative influence. Positive subjective task value is just as important as PE and EE in motivating learners’ intentions to continue using web-based learning (Chiu and Wang, 2008).

In sum, these studies stressed the significant influence of the UTAUT model in predicting and explaining the usage behavior across various digital technology domains.

### 2.3 ECM

In 2001, Bhattacherjee merged the Technology Acceptance Model (TAM) and Expectation Confirmation Theory (ECT) to create a new model for the continued utilization of information systems—the Expectation Confirmation Model (ECM). The ECM explains users’ post-adoption Use Behavior (UB) and the factors affecting their continued use intentions from aspects like expectation confirmation, perceived usefulness, and satisfaction. Comparing the actual utility after initial training with the expected usage, expectation confirmation impacts users’ perceived usefulness and satisfaction, thus indirectly affecting their sustained use. Perceived usefulness can also have a direct positive influence on user satisfaction. User Satisfaction is a crucial factor in influencing perceived usefulness and sustained use, while expectation confirmation and perceived usefulness are key prerequisites for satisfaction (Bhattacherjee, 2001).

Currently, the application of ECM primarily centers on information technology, encompassing the utilization of mobile applications and online platforms (Tam et al., 2020), acceptance of mobile advertising (Lu et al., 2019), and use of smart devices (Pal et al., 2020). Researchers often explore users’ satisfaction and their continued intent and behavior towards different technologies, products, or services by adjusting variables or integrating other models based on their research conditions. Jung-Chieh Lee and others used ECM to study how AI functions influence user’s continued intention to use mobile banking applications. The study indicated that both AI and anthropomorphic services can enhance user satisfaction by improving expectation confirmation and perceived usefulness, thus promoting continued mobile banking use (Lee et al., 2023). Neeraj Dhiman integrated the Task-Technology Fit (TTF) model with ECM to delve into the continued intent of users to use service chatbots during travelling. The results showed that when users find the technical characteristics of chatbots fit their tasks, their expectations are confirmed and directly impact their perceived usefulness. Both perceived usefulness and expectation confirmation positively influence user satisfaction towards chatbots, with perceived usefulness having the stronger impact (Neeraj and Jamwal, 2022). Baker-Eveleth used ECM to investigate students’ continued intent to use e-textbooks. The findings showed that students’ willingness to use e-textbooks is driven by satisfaction and perceived usefulness. Additionally, students’ expectation confirmation and the availability of e-textbooks have a positive impact on satisfaction and perceived usefulness, thus influencing the intention to continue using e-textbooks (Lori and Stone, 2015).

### 2.4 Hypothesize development

AI chatbots, considered landmarks in the new era of AI, has attracted considerable attention for its potential to revolutionize AI education. It is crucial to examine the factors that contribute to the sustained usage of AI chatbots by users, which has emerged as an important research inquiry. Media reports have highlighted the integration of AI chatbots in higher education institutions, providing valuable support to students in various academic activities such as essay writing assistance and question answering (Zhu and Wang, 2023). Consequently, investigating the determinants of AI chatbots adoption among university students, especially graduate students, holds paramount significance in this field.

Previous research has extensively utilized the UTAUT model to empirically examine the factors that influence users’ BI. These studies have established that the UTAUT model offers a more precise and effective explanation of users’ adoption and utilization behaviors towards technology (Holden and Karsh, 2009). For instance, El-Masri and Tarhini’s (2017) study on the acceptance of e-learning systems reveals a significant association between PE and BI. Similarly, Bao (2017) demonstrates that PE positively influences the adoption of mobile learning by open educational learners, while EE negatively impacts their willingness to adopt it. Wang and Mao (2016) assert that PE and EE serve as influential determinants affecting students’ engagement in online learning.

Building upon these findings, Lori and Stone (2015) highlight the considerable impact of PE, EE, and FC on the formation of students’ BI. These factors collectively shape students’ perceptions and attitudes towards online learning platforms. Meanwhile, El-Masri and Tarhini (2017) emphasize the crucial mediating role of “EE” in students’ intentions to continue using electronic learning systems, thus enhancing our understanding of the factors influencing sustained usage. Hu et al. (2020) and Raza et al. (2022) further support the critical influence of “EE” within the contexts of mobile learning and learning management systems, underscoring its significant impact on user acceptance and system adoption.

Furthermore, Zhang et al. (2023) provide compelling evidence of the causal relationship between social factors and users’ intention to adopt and utilize technology-mediated learning systems, thereby advancing our understanding of the intricate interplay between sociocultural elements and users’ BI. Wu et al. (2022) posit a positive correlation between variables of PE, SI, and FC with the adoption of AI-assisted learning among university students. Similarly, Zhai et al. (2022) proposes that FC indirectly impact the sustained usage intention of AI-assisted learning. Additionally, Zhang et al. (2016) examine the behavioral usage patterns of teachers in online learning environments, revealing significant influences of PE, EE, and SI on their sustained usage, with EE not substantially affecting their usage intention. In the domain of mobile learning, Wang et al. (2009) and Donaldson (2010) investigate the factors influencing users’ acceptance of mobile learning, emphasizing the importance of PE, EE, and SI. Moreover, the role of gender and age as moderating variables in these associations has been emphasized. Various studies, such as those conducted by Nikolopoulou et al. (2020) in the realm of mobile learning, Samsudeen and Mohamed (2019) concerning e-learning platforms, and Ain et al. (2016) focusing on learning management systems, underscore the significance of FC as determinant of learners’ BI and UB. These factors play a critical role in individual technology usage. Additionally, in the context of mobile learning (Kang et al., 2015), e-learning platforms, and augmented reality, FC has been recognized as pivotal factors influencing the adoption of diverse educational technologies in higher education. Furthermore, Wan and Zhao (2016) have identified substantial impacts of both FC and BI on UB. Drawing upon the insights, this study formulates the following hypotheses to examine the causal relations between PE, EE, SI, FC and BI when AI Chatbots are used:

*H1:* PE has direct and significant impact on BI.

*H2:* EE has direct and significant impact on BI.

*H3:* SI has direct and significant impact on BI.

*H4:* FC has direct and significant impact on BI.

*H5:* FC has direct and significant impact on UB.

*H12:* BI has direct and significant impact on UB.

The concept of “Satisfaction (SA)” in the context of information technology usage pertains to the subjective evaluation by users, reflecting their overall contentment with the technology (Gan and Wang, 2015). Notably, Gan and Wang (2015) reveals a strong positive correlation between satisfaction and sustained UB. This finding aligns with the work of Cidral et al. (2018), who emphasize the crucial role of SA as a fundamental construct for assessing the long-term adoption and continued usage of electronic learning systems. Furthermore, Zhang and Bai (2017) assert the prominent influence of SA as a critical factor impacting users’ enduring engagement with such systems. Overall, these scholarly insights underscore the significance of SA as a key determinant shaping users’ sustained UB in the context of information technology adoption.

In ECM, Confirmation (*CF*) is defined as “the users’ level of the appropriateness between their actual performance and expectation of the usage of information systems and services” (Hsu and Lin, 2015). ECM posits that users’ perceived “Confirmation (*CF*)” indirectly influences their BI by impacting factors of SA and “Perceived ease of use (PEU)” (Bhattacherjee, 2001). Notably, within this study, the selected variable representing “PEU” aligns with the concept of “PE” in the UTAUT model. This deliberate choice enhances the theoretical continuity and integration between the Technology Acceptance Model (TAM) and the UTAUT model, ensuring a comprehensive examination of user behavior in the present research context.

Li and Zhao (2016) highlight the pivotal roles of *CF* and SA in shaping UB. Zhu (2017) conducted a rigorous empirical study in the domain of online learning spaces, yielding significant findings regarding the relationship between SA and students’ intention to maintain their usage. This study demonstrates the influential role of SA in fostering a positive inclination among students to continue utilizing online learning platforms. Expanding on these foundations, Bhattacherjee (2001) emphasizes the significant impact of *CF* on user SA, thus indirectly affecting their sustained usage. Additionally, Yang et al. (2015) also underscores the consequences of *CF* on both user satisfaction and the intention to continue using. It is not clear whether those findings apply for the adoption of AI Chatbots. Accordingly, the following hypotheses were proposed:

*H6:* SA positively influences BI.

*H7:* PE positively influences SA.

*H8:* EE positively influences SA.

*H9: CF* positively influences PE.

*H10: CF* positively influences SA.

“Personal innovativeness (PI)” refers to an inherent characteristic that reflects an individual’s cognitive orientation and propensity for action (Xiong, 2015). Notably, Agarwal and Karahanna (2000) introduced the concept of PI in the context of information technology to shed light on users’ willingness to adopt novel technological advancements. Subsequent research in various information technology domains consistently acknowledges the significant influence of personal innovativeness on users’ acceptance of emerging technologies. For example, Twum et al. (2022) demonstrate the substantial impact of personal innovativeness on users’ adoption of electronic platforms, while Dajani and Abu Hegleh (2019) emphasize its pivotal role as a determinant in university students’ utilization of animation. Additionally, Sitar-Taut and Mican (2021) highlight the distinct significance of PI in the domain of mobile learning.

Within the higher education context, personal innovativeness serves as a crucial supplementary construct in the UTAUT model. The UTAUT model posits that user characteristics, such as gender, age, experience, and voluntariness, act as influential moderators across the core dimensions of acceptance and use behavior. Considering the specific context of the present study where participants engaged voluntarily with AI Chatbots and possessed limited experience with the technology, the moderator variables of voluntariness and experience were excluded. Building upon these considerations, the present study proposes the following hypothesis:

*H11:* Personal innovativeness has positive impact on BI.

**Figure 1:**
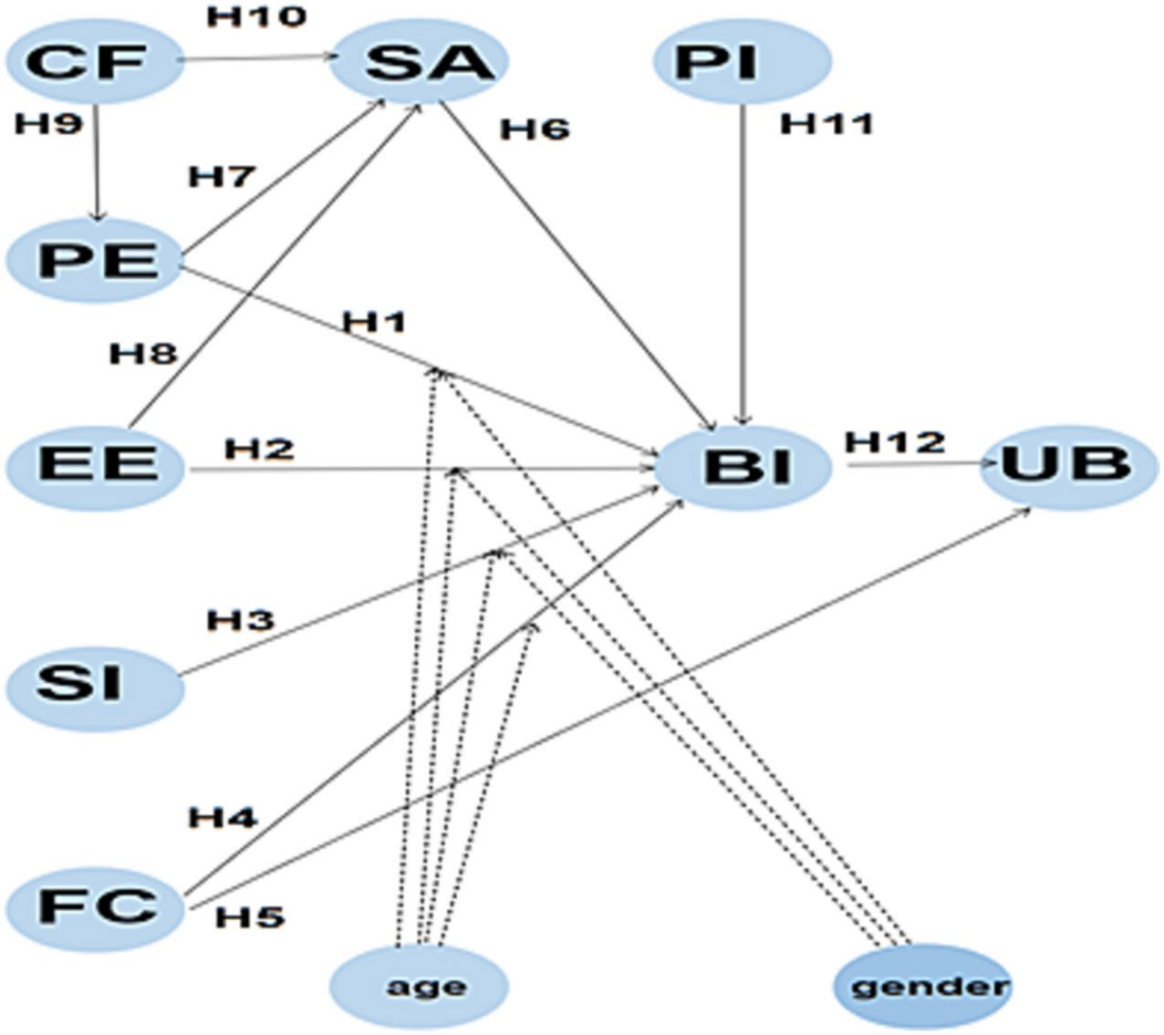
Research model hypothesis.

Figure 1 displays the study’s hypothesized model, which includes seven predictors, four of which are originally from UTAUT model, two from ECM model, and an additional external variable, “Personal innovativeness.”

## 3 Materials and methods

### 3.1 Data collection

The survey encompassed master’s and doctoral students from various Chinese universities like Xinjiang University, Ocean University of China, Dalian University of Science and Technology. It was conducted via the “Wenjuanxing” platform from May 30, 2023, and extended for two weeks. The survey was administered three times to different cohorts of graduate students, resulting in the collection of 637 questionnaires. After data cleaning, 373 valid responses remained (only those completed in at least 100 s and with distinct scores for all items were retained), yielding an effective response rate of 58.6%.

Table 1 presents the demographic characteristics of the study participants. Out of the 373 respondents, there were 193 males and 180 females. The majority of participants were master’s students, with an average age range of 20–25 years. Master’s students constituted 84.2% of the total graduate student population, while the ratio of master’s to doctoral students was approximately 5.3:1. Comparing this with data from the National Bureau of Statistics of China (NBSC) (2022), which reported that master’s students comprised about 84.7% of the graduate student population in 2021, with a master’s to doctoral student ratio of approximately 5.5:1 (National Bureau of Statistics of China (NBSC), 2022), it suggests that the sampling distribution in our study aligns well with the actual population and demonstrates strong representativeness.

**Table 1:** Respondents’ characteristics.

Classification	Frequency	Percentage (%)
Male	193	51.7
Female	180	48.3
Under 20 years old	4	1.1
20–25 years old	232	62.2
26–30 years old	105	28.2
31–35 years old	21	5.6
Above 35 years old	11	2.9
Master	314	84.2
Doctoral	59	15.8

### 3.2 Instruments

The research instrumentation in this study consisted of two components. The first component captured demographic information such as participants’ cities, gender, ages, and educational background. The second component utilized a seven-point Likert scale, ranging from “1 = strongly disagree/unsatisfied” to “7 = strongly agree/satisfied,” to collect responses. This scale aimed to assess the factorial structure of UTAUT and ECM in relation to Chinese graduates’ willingness to utilize an AI chatbot. To ensure consistent estimation, we established a numerical metric scale from 1 to 7, associating specific frequencies with each point on the scale. This scale is defined as follows: “Never” corresponds to 1, “Once a month” corresponds to 2, “Several times a month” corresponds to 3, “Once a week” corresponds to 4, “Several times a week” corresponds to 5, “Once a day” corresponds to 6, and “Several times a day” corresponds to 7.

In the second part of the instrumentation, a total of 29 items were developed by drawing from previous research studies conducted by Agarwal and Prasad (1998), Bhattacherjee (2001), Venkatesh et al. (2003), Lee (2010), Venkatesh and Xu (2012), and Yang (2016). These items covered various constructs, including “PE” (four items), “EE” (three items), “SI” (three items), “FC” (four items), “*CF*” (three items), “SA” (four items), “PI” (four items), “BI” (three items), and “UB” (one item). To ensure linguistic equivalence, a forward-backward translation procedure was employed, following the method suggested by Li et al. (2019) and Brislin (1986). Firstly, an English teacher translated the items into Chinese, and then another English teacher back-translated the Chinese version into English. Any discrepancies in the translations were resolved through negotiation and adjustments in wording. The accuracy of the Chinese translations was confirmed by the high similarity observed between the two versions. For detailed information on the measurement scale and descriptive statistics, please refer to Table 2.

**Table 2:** Measurement scale and factor loadings.

Construct	Item	Items	Items in Chinese	Loading	Mean	St.dev.	Adapted from
Performance expectancy	PE1	“I believe that AI Chatbot is useful in my studies”	“我认为AI聊天机器人对我的学习/研究非常有用”	0.854	5.03	1.338	Venkatesh et al. (2003), Venkatesh and Xu (2012)
	PE2	“Using AI Chatbot increases my chances of achieving important things in my studies”	“AI聊天机器人可以增加我在学习/研究中获得更高成就的机会”	0.845	4.76	1.45	
	PE3	“Using AI Chatbot helps me get tasks and projects done faster in my studies”	“AI聊天机器人可以帮助我更快地完成学习任务和研究项目”	0.836	5.04	1.315	
	PE4	“Using AI Chatbot increases my productivity in my studies”	“AI聊天机器人可以让我更方便地咨询、解决学习问题，提升学习效率”	0.788	5.21	1.288	
Effort expectancy	EE1	“Learning how to use AI Chatbot is easy for me”	“对我来说，学习使用AI聊天机器人很容易，没有什么难度”	0.796	5.03	1.31	Venkatesh et al. (2003), Venkatesh and Xu (2012)
	EE2	“My interaction with AI Chatbot is clear and understandable”	“对我来说，AI聊天机器人反馈的信息非常清晰，容易理解”	0.855	4.93	1.349	
	EE3	It is easy for me to become skillful at using AI Chatbot”	“我认为，我很快就可以精通AI聊天机器人产品”	0.744	4.85	1.37	
Social influence	SI1	“People who are important to me think I should use AI Chatbot”	“对我非常重要的人，让我觉得应该使用AI聊天机器人”	0.858	4.63	1.566	Venkatesh et al. (2003), Venkatesh and Xu (2012)
	SI2	“People who influence my behavior believe that I should use AI Chatbot”	“对我行为有影响力的人，让我觉得自己应该使用AI聊天机器人”	0.895	4.45	1.565	
	SI3	“People whose opinions I value prefer me to use AI Chatbot”	“我重视其意见的人，更希望我使用AI聊天机器人”	0.888	4.37	1.519	
Facilitating conditions	FC1	“I have the resources necessary to use AI Chatbot”	“我拥有使用AI聊天机器人所必备的资源条件”	0.759	4.64	1.427	Venkatesh et al. (2003), Venkatesh and Xu (2012)
	FC2	“I have the knowledge necessary to use AI Chatbot”	“我拥有使用AI聊天机器人所必备的知识背景”	0.837	4.68	1.398	
	FC3	“AI Chatbot is compatible with technologies I use”	“我拥有使用AI聊天机器人所必备的技术条件”	0.891	4.79	1.325	
	FC4	“I can get help from others when I have difficulties using AI Chatbot”	“当我在使用 AI聊天机器人遇到困难时，我可以比较容易地从他人那里获得帮助”	0.679	4.85	1.393	
Confirmation	CF1	“My usage of AI chatbots for learning and research has surpassed my expectations.”	“我使用AI聊天机器人学习、科研的经历比我预期的要好”	0.856	4.99	1.423	Bhattacherjee (2001), Lee (2010)
	CF2	“The learning and research materials offered by AI chatbots have surpassed my initial expectations.”	“AI聊天机器人提供的学习、科研内容水平比我预期的要好”	0.857	4.84	1.497	
	CF3	“Overall, I have found that my expectations regarding the use of AI chatbots have been largely met.”	“总体来说，我对使用AI聊天机器人的预期大都得到了满足”	0.838	4.76	1.432	
Satisfaction	SA1	“I believe that using AI chatbots for learning and research is a wise decision.”	“我认为使用AI聊天机器人学习、科研的决策是明智的”	0.842	4.79	1.454	Bhattacherjee (2001), Yang (2016)
	SA2	“I find the experience of using AI chatbots for learning and research to be enjoyable.”	“我认为使用AI聊天机器人学习、科研的经历是愉快的”	0.903	5.01	1.317	
	SA3	“I am satisfied with the effectiveness of using AI chatbots for learning and research.”	“我对使用AI聊天机器人学习、科研的效果是满意的”	0.879	4.86	1.335	
	SA4	“Overall, I am satisfied with using AI chatbots for learning and research.”	“总体来说，我对使用AI聊天机器人学习、科研感到满意”	0.892	4.95	1.288	
Personal innovativeness	PI1	“I like experimenting with new information technologies mentally and operationally”	“无论是想法还是行动上，我愿意体验、接受新技术”	0.753	5.49	1.189	Agarwal and Prasad (1998)
	PI2	“If I heard about a new information technology, I would look for ways to experiment with it”	“一旦我听说出现了某项新技术，我会想方设法、排除困难去体验它”	0.827	5.03	1.366	
	PI3	“Among my family/friends, I am usually the first to try out new information technologies”	“与周围的家人、朋友相比，我总是更早地接触、尝试新技术”	0.813	5.24	1.247	
	PI4	“In general, I do not hesitate to try out new information technologies”	“总的来说，我总是充满好奇心，会毫不犹豫地尝试新的信息技术”	0.864	5.11	1.336	
Behavioral intention	BI1	“I intend to continue using AI Chatbot in the future”	“在未来，我会继续使用AI聊天机器人”	0.823	5.35	1.195	Venkatesh and Xu (2012)
	BI2	“I will always try to use AI Chatbot in my studies”	“在我的学习和研究中，我会一直使用AI聊天机器人”	0.892	4.95	1.319	
	BI3	“I plan to continue to use AI Chatbot frequently”	“在未来，我会非常频繁地使用AI聊天机器人”	0.847	4.76	1.365	
Use behavior	UB1	“Please choose your usage frequency for AI Chatbot: Never; Once a month; Several times a month; Once a week; Several times a week; Once a day; Several times a day”	请选择使用AI聊天机器人学习、科研的频率:从不；一月一次；一月多次；一周一次；一周多次；一天一次；一天多次	1.000	3.86	1.675	Venkatesh and Xu (2012)

To facilitate students’ understanding, we inform the survey participants that AI chatbots may include products such as ChatGPT, Bing, Notion AI, Wenxin Yiyan, etc.

### 3.3 Data analysis procedure

Data analysis in this study was conducted using the Statistical Package for the Social Sciences (SPSS) and the PLS-SEM (Partial Least Squares Structural Equation Modeling) method. PLS-SEM combines principal component analysis with ordinary least squares regression and was employed to estimate the hypothesized study model. The selection of PLS-SEM was based on two reasons: First, the study adopted a composite-based model for theory development, which is both causal and predictive (Hair et al., 2021). Second, PLS-SEM is robust to data distribution assumptions, making it suitable for analysis in this study (Hair et al., 2021).

The PLS-SEM entails two main phases: the measurement model and the structural model. The measurement model establishes the relationships between latent variables (unobserved variables) and measurement variables (observed variables) to explain causal relationships (Wang and Cao, 2020). PLS-SEM, a non-parametric structural equation modeling method, is suitable for non-normal, small sample, and exploratory studies. It does not strictly assume sample distribution and handles complex structural equation models. A minimum sample size of 10 times the number of indicators is recommended (Haenlein and Kaplan, 2004; Hair et al., 2021).

With 373 valid samples and 29 items, the sample-to-indicator ratio in this study met the PLS-SEM requirement of at least a 10:1 ratio (Kock, 2018).

## 4 Result

### 4.1 Measurement model

In assessing the measurement model, this study conducted an evaluation of factor loadings, reliability, convergence validity, and discriminant validity, following the approach outlined by Hair et al. (2021). The assessment utilized Cronbach’s alpha, AVE (average variance extracted), and CR (composite reliability) as reliability indicators, in line with the guidance provided by Bagozzi and Yi (1988). These metrics were computed using the PLS Algorithm in Smart PLS, with the results detailed in Table 3. Reliability serves as an indicator of the questionnaire’s consistency and stability, with higher values signaling greater questionnaire reliability and internal consistency.

**Table 3:** The reliability and convergent validity of the research constructs.

Variables	Cronbach’s alpha	Composite reliability	Average variance extracted (AVE)
BI	0.815	0.890	0.730
*CF*	0.818	0.892	0.733
EE	0.717	0.842	0.640
FC	0.802	0.872	0.633
PE	0.851	0.899	0.690
PI	0.831	0.888	0.665
SA	0.902	0.932	0.773
SI	0.855	0.912	0.775

Cronbach’s alpha is commonly used as a measure of overall scale reliability, with a value greater than 0.70 considered acceptable. A higher Cronbach’s alpha coefficient indicates a higher level of reliability (Hair et al., 2021). According to Fornell and Larcker (1981), AVE (average variance extracted) value greater than 0.50 is considered ideal, while a range of 0.30–0.50 is deemed acceptable. When both AVE and CR exceed 0.50 and 0.70 respectively, it indicates good reliability of the factors. Table 3 presents the results, indicating that the lowest values for Cronbach’s alpha (0.717), AVE (0.633), and CR (0.842) in this study surpass the general standards (Cronbach’s alpha >0.70, AVE > 0.50, and CR > 0.70). Moreover, considering that the questionnaire was adapted from a mature model, it demonstrates a desirable level of reliability and consistency in its structure.

**Figure 2:**
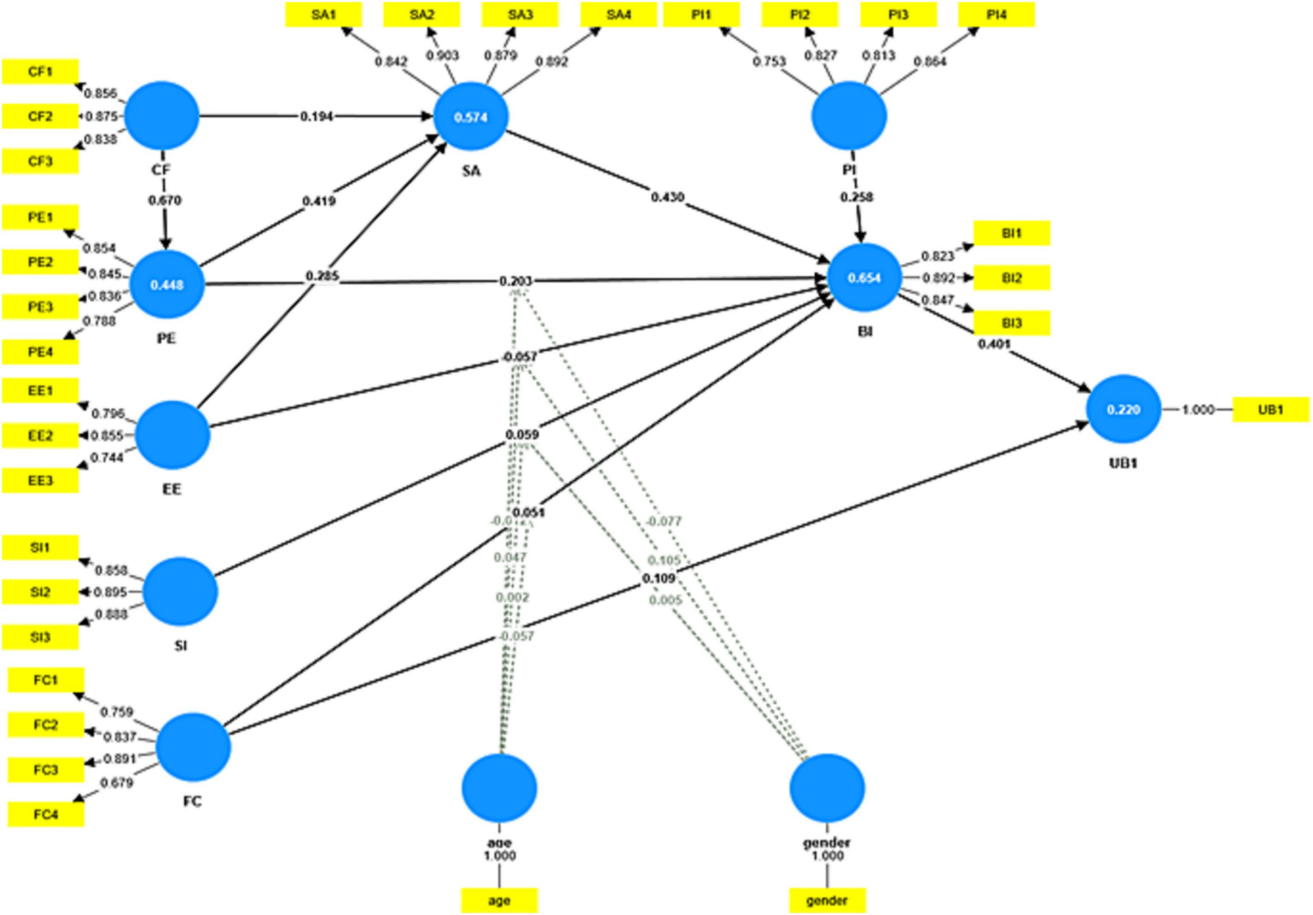
Measurement model with standardized factor load coefficients and path coefficients.

The questionnaire in this study has undergone meticulous development, drawing on empirical studies within the same thematic or disciplinary domain, which significantly contributes to its high content validity. Hair et al. (2021) emphasizes the importance of both convergent validity and discriminant validity. Convergent validity is established when factor loadings exceed 0.70, and it is considered acceptable when they exceed 0.50. From Figure 2 and Table 2, all factor loadings exceed 0.70, except for FC4, which exceeds 0.50, indicating high convergent validity. Additionally, Fornell and Larcker (1981) suggest an AVE value of at least 0.50 for convergent validity. As shown in Table 3, all AVE values surpass 0.50, further supporting high convergent validity.

Discriminant validity was assessed using the heterotrait-monotrait ratio of correlations (HTMT) proposed by Henseler et al. (2015). The HTMT threshold of 0.90 was used to evaluate effective discrimination. Table 4 shows that all item measurement values are below 0.90, indicating good discriminant validity.

**Table 4:** HTMT values.

	BI	*CF*	EE	FC	PE	PI	SA	SI	UB1
BI									
*CF*	0.649								
EE	0.677	0.635							
FC	0.683	0.643	0.705						
PE	0.789	0.794	0.627	0.627					
PI	0.793	0.586	0.690	0.668	0.675				
SA	0.875	0.711	0.727	0.701	0.784	0.695			
SI	0.534	0.497	0.592	0.578	0.567	0.366	0.567		
UB1	0.509	0.296	0.345	0.365	0.409	0.355	0.444	0.232	

BI, behavioral intention; CF, confirmation; EE, effort expectancy; FC, facilitating conditions; PE, performance expectancy; PI, Personal innovativeness; SA, satisfaction; SI, social influence; UB, use behavior.

### 4.2 Structural model

Through the higher-order structural model, we examined the causal relationships between latent variables and evaluated the model’s predictive power. To assess this, we employed various measures such as the variance inflation factor (VIF), path coefficients, f-squared, and R-squared (Stone, 1976). The model’s path parameters were estimated, and the significance of the path coefficients was evaluated using the Bootstrapping algorithm (Chin, 1998) with 5,000 resamples from valid data, as shown in Table 5. Additionally, we tested the moderation effects of “gender” and “age” in the model and assessed their significance, as presented in Table 6. Collinearity was assessed using VIF, with a criterion of VIF values less than 5 for all endogenous variables, as outlined by Hair et al. (2021). In our analysis, all path VIF values were below 5, indicating the absence of collinearity, as shown in Table 7.

**Table 5:** Path coefficients and the results of the significance tests.

H	Path	β	*t*-value	*p* value	Decision
H1	PE → Behavioral intention	0.203	2.917	0.004	Supported
H2	Effort expectancy → Behavioral intention	−0.057	0.935	0.350	Rejected
H3	Social influence → Behavioral intention	0.059	0.874	0.382	Rejected
H4	Facilitating Conditions → Behavioral intention	0.051	0.909	0.364	Rejected
H5	Facilitating Conditions → Use behavior	0.109	1.949	0.051	Rejected
H6	Satisfaction → Behavioral intention	0.059	6.408	0.000	Supported
H7	Performance expectancy → Satisfaction	0.419	8.532	0.000	Supported
H8	Effort expectancy → Satisfaction	0.285	6.289	0.000	Supported
H9	Confirmation → Performance expectancy	0.670	20.144	0.000	Supported
H10	Confirmation → Satisfaction	0.194	4.065	0.000	Supported
H11	Personal innovativeness → Behavioral intention	0.258	5.126	0.000	Supported
H12	Behavioral intention → Use behavior	0.401	7.327	0.000	Supported

**Table 6:** Moderating effects.

Path	β	*T*-value	*p* value	Moderating effect
Age × Performance expectancy → Behavioral intention	−0.012	0.283	0.777	No
Age × Effort expectancy → Behavioral intention	0.047	1.240	0.215	No
Age × Social influence → Behavioral intention	0.002	0.048	0.962	No
Age × Facilitating Conditions → Behavioral intention	−0.057	1.286	0.199	No
Gender × Performance expectancy → Behavioral intention	−0.077	0.708	0.479	No
Gender × Effort expectancy → Behavioral intention	0.105	1.125	0.261	No
Gender × Social influence → Behavioral intention	0.005	0.058	0.954	No

**Table 7:** Result of the variables’ collinearity indicators and the intensity of the effect.

Path	f^2^	VIF
Performance expectancy → Behavioral intention	0.034	3.517
Performance expectancy → Satisfaction	0.212	1.937
Effort expectancy → Behavioral intention	0.002	3.854
Effort expectancy → Satisfaction	0.136	1.404
Social influence → Behavioral intention	0.003	3.661
Facilitating Conditions → Behavioral intention	0.004	1.944
Facilitating Conditions → Use behavior	0.011	1.437
Satisfaction → Behavioral intention	0.201	2.653
Confirmation → Performance expectancy	0.813	1.000
Confirmation → Satisfaction	0.046	1.931
Behavioral intention → Use behavior	0.143	1.437
Personal innovativeness → Behavioral intention	0.094	2.045

Table 7 provides statistical confirmation for each path in the model. The f-squared analysis was used to estimate the strength of the relationships between variables, with values greater than 0.02, 0.15, and 0.35 indicating small, medium, and large effects, respectively (Hair et al., 2021). The table also displays collinearity results, showing the relationships between endogenous variables and their regression weights with exogenous variables (path coefficients), indicating the presence of multicollinearity among the indicators.

Table 8 evaluates the model’s accuracy using the coefficient of determination, R-squared, which measures the explanatory power of the model. Ranging from 0 to 1, a higher value indicates stronger explanatory power, where 0.75, 0.50, and 0.25 are considered substantial, moderate, and weak, respectively (Hair et al., 2021). The endogenous variables BI (R^2^ = 0.639), PE (R^2^ = 0.447), and SA (R^2^ = 0.570) exhibit strong explanatory power, while UB1 (R^2^ = 0.216) has weaker explanatory power. Q-squared values are employed to assess the impact of exogenous variables on endogenous constructs (Stone, 1976). Q-squared values greater than 0.02, 0.15, and 0.35 indicate small, medium, and large predictive relevance, respectively (Hair et al., 2021). As shown in Table 8, the endogenous variables BI (Q^2^ = 0.453), PE (Q^2^ = 0.444), SA (Q^2^ = 0.469), and UB1 (Q^2^ = 0.131) demonstrate strong predictive capability. The model fit was assessed using the Goodness of Fit (GOF) proposed by Tenenhaus et al. (2004) and the Standardized Root Mean Square Residual (SRMR) proposed by Henseler and Sarstedt (2012). Both GOF and SRMR were used to evaluate the model’s fit, as researchers have debated their relative importance (Henseler and Sarstedt, 2012).

**Table 8:** The predictive power of structural model.

	Q^2^	R^2^
BI	0.453	0.639
PE	0.444	0.447
SA	0.469	0.570
UB1	0.131	0.216
GOF= $\sqrt{\bar{\mathrm{communilities}}\times \bar{R²}}$ = $\sqrt{0.37425\times 0.468}$ ≈0.419 SRMR = 0.052		

Table 8 provides the GOF values and SRMR values to evaluate the model fit. GOF values of 0.01, 0.25, and 0.36 are considered small, medium, and large, respectively. For this study, the computed GOF value is approximately 0.419, indicating a good overall fit of the model. The SRMR, which should be less than 0.08 for a good fit, is calculated as 0.052 in this study.

**Figure 3:**
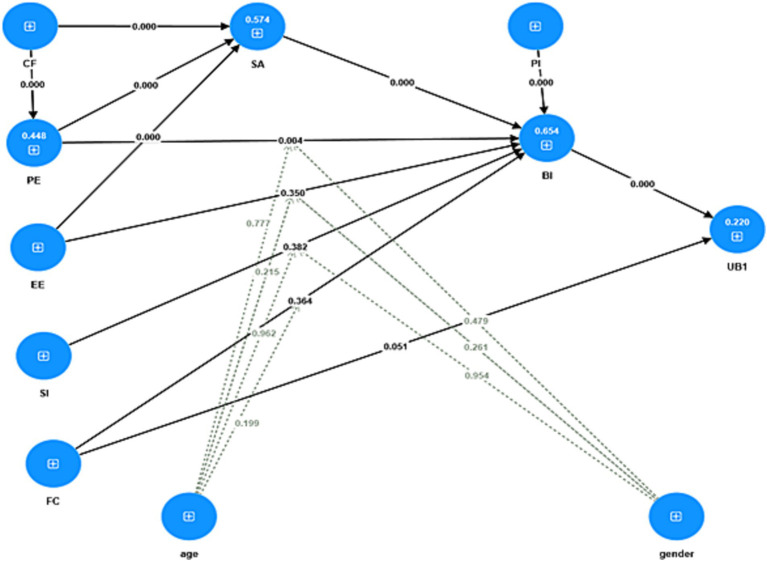
The structural model with path coefficients.

**Figure 4:**
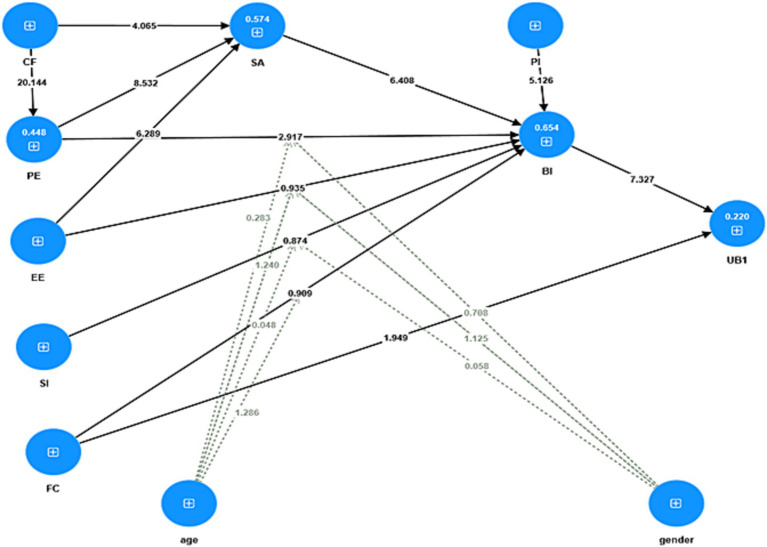
The structural model with *t*-values.

Confirmation of hypotheses relies on the *t*-values and *p*-values presented in Table 5. A hypothesis can be confirmed if the *p*-value is ≤0.05 and the *t*-value is ≥1.96. Based on Table 5, eight hypotheses (H1, H6, H7, H8, H9, H10, H11, H12) are supported, while the remaining four hypotheses (H2, H3, H4, H5) are not supported. The path coefficients for the structural model are shown in Figure 3, and the t-values for the structural model are displayed in Figure 4.

## 5 Discussion

Our study investigates the integration of AI Chatbots among Chinese postgraduate students, merging the UTAUT and ECM models. Despite the limited prior exploration of this subject, particularly in higher education settings, our findings hold significant implications for advancing the understanding of AI chat technologies as educational tools. Notably, our results confirm a positive correlation between “Performance Expectancy (PE)” and “Behavioral Intention (BI)” among postgraduates regarding AI Chatbot usage, aligning with the observations made by El-Masri and Tarhini (2017) in their study of e-learning system acceptance. This positive association between “PE” and “BI” has been consistently observed in various domains, including learning management software (Kang et al., 2015), mobile learning (Kumar and Bervell, 2019), and online learning (Chiu and Wang, 2008). Previous research consistently identifies “PE” as a critical predictor of “BI.”

This substantive association between “PE” and “BI” underscores the critical role of performance expectancy in shaping the behavioral intention towards technology adoption, a pattern evident across diverse educational realms. Understanding this relationship within the context of AI chatbot integration among postgraduate students not only provides insights into their acceptance, but also highlights the influential role of user experience in driving their intention to utilize AI chatbots as educational resources. Moreover, this analysis emphasizes the transferability of these insights from prior technological adoption studies to the domain of AI chatbot implementation within higher education, signifying the overarching significance of performance expectancy in shaping behavioral intent within educational technology adoption.

Meanwhile, our examination reveals that “Effort Expectancy (EE)” demonstrates no statistically significant impact on “Behavioral Intention (BI)” of postgraduates on using AI Chatbot for academic research, aligning with the findings of EE on BI reported by Bao (2017). This indicates that the adoption of AI Chatbots in higher education necessitates minimal effort without significantly influencing users’ intention to utilize them. Additionally, a substantial positive relationship is observed between “Performance Expectancy (PE)” and “Effort Expectancy (EE)” with “Satisfaction (SA).” Specifically, the support offered by AI Chatbots to graduate students in their research and learning endeavors, along with their comprehension of the feedback received, impacts the levels of satisfaction following actual usage. “Satisfaction” emerges as the primary and most influential predictor of “BI.”

Furthermore, our study unveils that both “Social Influence (SI)” and “Effort Expectancy (EE)” do not exert a significant impact on “Behavioral Intention (BI),” delineating insignificant effect sizes and deviating from the UTAUT model. The lack of substantial influence from “Social Influence (SI)” on the adoption of AI Chatbots by graduate students can be attributed to two primary factors. First, this outcome is likely associated with the heightened educational background of the participants, indicative of a higher level of discernment and reduced susceptibility to external influences. Second, it can be ascribed to the relatively limited usage and uptake of AI Chatbot technology among the target population in China, which has not gained widespread acceptance and recognition in society.

This observation aligns with previous studies (Kumar and Bervell, 2019; Alotumi, 2022), which similarly reported no significant impact of “Social Influence (SI)” on users’ sustained usage. These findings collectively underscore the complex interplay of factors influencing the adoption of AI Chatbots in higher education and pave the way for a deeper understanding of the nuanced dynamics at play. Further exploration of these layered dynamics can provide essential insights for refining the implementation and utilization of AI Chatbots within the educational landscape.

In addition, the impact of “Facilitating Conditions (FC)” on users’ sustained usage of AI Chatbots is not found to be statistically significant. This can be attributed to the perceived ease of use and comprehension among graduate students, who belong to a generation well-versed in internet technology, when interacting with AI Chatbots. Simple prompts suffice for them to complete tasks without necessitating additional resources or equipment. Consequently, “FC” does not significantly influence users’ intention for continued use. Our study also reaffirms the noteworthy impact of “Satisfaction” on “Behavioral Intention (BI)” of postgraduates on using AI Chatbots, in line with earlier research (Gan and Wang, 2015; Li and Zhao, 2016) of “Satisfaction” on “Behavioral Intention (BI).”

Moreover, “Confirmation (*CF*)” positively influences both “Performance Expectancy (PE)” and “Satisfaction (SA)” on graduate students using AI Chatbots on research. Within the ECM model, the level of confirmation users receive regarding their expectations before usage assumes a critical role in shaping their satisfaction. Greater confirmation leads to heightened satisfaction, subsequently driving continued usage. Notably, in our investigation, “*CF*” emerges as the primary and most influential predictor of “SA.” Additionally, “Personal Innovativeness (PI)” exhibits a significant influence on “BI” while serving as the second most influential predictor. AI Chatbots represent not only an innovation in traditional learning methods but also a technological innovation, positioning “PI” as a crucial individual difference factor.

Regarding moderating variables, our analysis indicates that the connections between the predictors and the dependent variable are not substantially impacted by the moderating variables of “Gender” and “Age.” This indicates the robustness and consistency of the observed patterns in influencing users’ behavioral intentions towards AI Chatbots, irrespective of gender and age, underlining the broad applicability and reliability of the identified predictors in shaping user intent and behavior. This comprehensive understanding of the multifaceted factors and their interplay not only enriches our comprehension of AI chatbots adoption but also offers valuable insights for tailoring strategies to optimize their effective implementation within higher education settings.

## 6 Conclusion

In our current investigation, we have empirically verified 12 hypotheses and revealed a robust relationship between *CF*, SA, and BI in the ECM model, surpassing the strength of the relationship between UTAUT constructs and BI. This suggests that users of information technology, notably AI Chatbots as examined in our study of Chinese graduate students, engage in decision-making processes akin to consumer decisions for repeated consumption and purchases in marketing. The alignment between users’ expectations of an AI Chatbot and their initial experience significantly impacts their decision and intention to continue usage. Moreover, users’ actual satisfaction with an AI Chatbot is pivotal in shaping their subsequent intentions. This highlights the parallel mechanisms between consumer behavior and technology acceptance, emphasizing the relevance of user experience and satisfaction in shaping continued usage intentions.

### 6.1 Theoretical contribution

The existing literature predominantly focuses on the future development of AI Chatbots and the industry’s response to their impact, particularly ChatGPT. However, there is a dearth of research examining students’ perspectives and acceptance of AI Chatbots, specifically in China. Students express a strong desire for the integration of AI Chatbots in higher education, expecting them to positively impact their educational development. Some researchers have conducted interviews with ChatGPT (Lund and Wang, 2023) to explore its influence on education. The utilization of AI Chatbots in academia and higher education is relatively new and still in the exploratory stage. In this study, we adopted the UTAUT model as the foundational framework, while also incorporating elements from the ECM to further enhance our evaluation of Chinese graduate students’ attitudes and intentions towards using AI Chatbots.

Interestingly, we found that certain items within the UTAUT model inadequately captured the perspectives of Chinese graduate students on the acceptance and utilization of AI Chatbots. Conversely, constructs such as “Confirmation” and “Satisfaction” from the ECM model demonstrated better efficacy in assessing the technological acceptance levels among Chinese graduate students, particularly their perceptions of AI Chatbots. Notably, “Satisfaction” and “Personal innovativeness” emerged as critical factors significantly influencing Chinese graduate students’ intentions to accept and utilize AI Chatbots. It is essential to highlight the scarcity of previous research on the adoption and acceptance of AI Chatbots in Chinese higher education, underscoring the novelty and significance of our study. As a result, the findings from this study will contribute to enhancing our understanding of AI Chatbot deployment in higher education and facilitating the advancement and optimization of AI Chatbot applications within educational contexts.

### 6.2 Practical implications

The integration of AI chatbots in higher education pedagogy, as instrumental adjuncts in fostering research writing aptitudes, affords a multifarious linguistic repertoire and proffers critical feedback, thereby mitigating the anxieties surrounding academic writing endeavors (Guo et al., 2022). The study at hand substantiates that “Satisfaction” is a cardinal determinant in the behavioral intention (BI) of postgraduate scholars’ engagement with AI chatbots for research. To amplify scholarly rigor and assimilate AI chatbots seamlessly into the pedagogical milieu of Chinese graduate students, a pivot towards user-centric methodologies is imperative. Such approaches mandate an intimate acquaintance with academic exigencies and a relentless pursuit of strategically adaptive solutions.

Key considerations also include ensuring the authenticity and factual accuracy of interactions, ensuring data confidentiality, and exploring heightened collaboration between human intellect and computational systems to achieve a synergy of human-machine intelligence. The practical implications of these strategies for the educational sector, particularly within the investigated acceptance of AI Chatbots among graduate students for research and study, are multifaceted. Firstly, the emphasis on user-centric methodologies highlights the need for educational institutions to customize AI Chatbot interventions to correspond with students’ specific research and learning requisites. This underlines the importance of understanding postgraduates’ unique research demands, ensuring that AI Chatbots are tailored to enhance and streamline the research process, ultimately contributing to improved research outcomes and student satisfaction.

Moreover, the recognition of student satisfaction as a decisive factor in their intent to leverage AI Chatbots for research emphasizes the significance of AI Chatbots in enriching the research experience, thereby potentially enhancing academic productivity and knowledge creation. From a policy perspective, the practical implications necessitate the formulation of guidelines that promote ethical utilization of AI technologies, ensuring data privacy, and fostering transparent interactions. This underlines the imperative for educational policymakers to adopt a balanced approach that encourages innovation while prioritizing student well-being and ethical considerations. Additionally, fostering a regulatory framework that emphasizes the ethical and effective integration of AI technologies within the scholarly research landscape is pivotal in ensuring the responsible implementation of AI Chatbots in higher education.

### 6.3 Limitations

This study is not without limitations, all of which merit consideration. Firstly, it is essential to acknowledge the limitations of the UTAUT model, given that the majority of empirical research validating this model has been primarily conducted in developed countries. This raises the critical necessity for expanded empirical scrutiny to ascertain its applicability in distinct contexts, particularly in China, as underscored by Xiong (2015). Secondly, this study did not account for the respondents’ prior experience or duration of use with AI Chatbots, generating concerns regarding the uniformity and depth of users’ familiarity and engagement with the technology. Additionally, the geographical focus of the study primarily centered on provincial capitals and major cities, which potentially restricted the representation of perspectives from smaller cities. Furthermore, one must acknowledge that the level of economic development in these cities may significantly impact users’ acceptance and adoption of new technologies. To augment the scholarly rigor of future research in this burgeoning field, it is advisable to conduct a comprehensive assessment of the scales employed in this study and contemplate the integration of contextual factors to enhance the study’s validity and generalizability.

Considering the limitations, future research endeavors could delve into rectifying the identified shortcomings and further illuminating the understanding of AI Chatbot acceptance among graduate students for research and study purposes. Given the significance of contextual factors, subsequent research could aim to analyze the nuanced impacts of economic development and regional disparities on AI Chatbot acceptance within varied geographical settings in China. Additionally, delving into the role of prior experience and the duration of AI Chatbot use among respondents could elucidate the influence of familiarity and proficiency with the technology on their acceptance and utilization. Furthermore, future studies could endeavor to validate and refine the UTAUT model within the specific context of Chinese higher education, offering insights into its cross-cultural applicability and potential areas for modification or extension to suit the Chinese educational landscape. Through these future research efforts, the scholarly community would gain a more comprehensive understanding of AI Chatbot integration within scholarly settings, thereby refining pedagogical strategies and technological implementation to better serve graduate students’ academic needs.

## Data availability statement

The original contributions presented in the study are included in the article/supplementary material, further inquiries can be directed to the corresponding author.

## Author contributions

WT: Methodology, Project administration, Supervision, Writing – original draft, Writing – review & editing, Formal analysis, Funding acquisition, Investigation. JG: Methodology, Project administration, Supervision, Writing – original draft, Writing – review & editing, Conceptualization. YZ: Visualization, Writing – original draft. XZ: Visualization, Writing – review & editing.
